# Sub-Peritoneal Myoma*Reported to the Clinical Society.

**Published:** 1897-08

**Authors:** August Schachner

**Affiliations:** Louisville, Ky.; Demonstrator of Anatomy in the Louisville Medical College; Visiting Surgeon to the Louisville City Hospital, etc.


### Selections and Abstracts

### SUB-PERITONEAL MYOMA.*

By AUGUST SCHACHNER, M. D., Louisville, Ky.

Demonstrator of Anatomy in the Louisville Medical College; Visiting Surgeon to the Louisville City Hospital, etc.

The specimen which I present was exhibited at the last meeting of this Society, and before taking up the subject again I desire to say that I am sorry at the last meeting I was so poorly prepared as I was. Before presenting the specimen originally, I should have had a microscopic examination made, so that the subject might have been intelligently discussed.

For the benefit of those who were not present at the last meeting I will give a short history of the case: The patient was a woman aged about fifty years; married twice; had four children. When this tumor began its growth she does not know, but at the time I first examined her the enlargement reached almost from the pubes to the umbilicus, apparently quite fixed. Introducing my fingers into the vagina I felt a round, smooth mass about the size of my fist. On one side (the left) the fingers could be introduced some distance up the vagina, while on the right side the depth was much less; it was evident, however, by feeling, that there was a space above, although it could not be reached by the fingers. In sweeping the finger around this mass a constriction could be plainly made out as though it might be an inverted uterus, perhaps a fibroid tumor of the uterus that had grown downward carrying the uterus with it, and that the finger was being swept about the enlarged cervix. I made an examination with the speculum, and even then no trace of the cervix could be made out. Nothing could be found that would indicate the location of the uterus.

The patient was prepared for operation, everything being cleansed as thoroughly as possible. The abdominal route was chosen and as soon as the incision was made I came down upon a large mass which was fixed in the pelvis. I did not make a very careful examination at the time, but, having the abdomen open, immediately proceeded to try and enucleate the tumor. I grasped it with volsellum forceps, and after much manipulation and pulling succeeded in getting the tumor about half out of the abdomen, when the uterus was plainly apparent just

* Reported to the Clinical Society.

above the tumor, as I now demonstrate to you. The tumor was quite adherent at its posterior surface down to Douglas pouch, and hemorrhage was considerable, especially at one point, which rather confused things. I threw a rubber ligature around the tumor to control bleeding and proceeded to cut the growth away. Making a careful dissection behind the tumor I encountered a cavity which led to the further examination, and I finally concluded that I was in the vagina. The remaining attachments of the tumor were now severed and the delivery completed, the peritoneal cavity being carefully walled off from above. The vagina was easily brought up flush with the abdominal incision and, being anchored there by means of an hysterectomy pin, was sutured without trouble. The abdominal wound was closed above, which left a raw surface at the upper part of the vagina, which was packed with gauze. The gauze was changed at the end of twenty-four hours. For the first two or three days there was a considerable oozing.

In my former report I made one statement in particular which is not borne out by the microscopical examination. I said that I thought the hood or top of the tumor was composed of the vagina. Dr. Weidner, who made an examination with the microscope, says he does not believe it is the vagina. The vaginal wall, however, extends up some distance on the outside of the tumor, which is a point I tried to make clear in my previous report. The tumor evidently began its growth from beneath the peritoneum, above the mucous membrane of the vagina, possibly from behind the uterus, or it may be that it had no connection whatever with the uterus. I am free to confess that it would be hard to determine from what structure the tumor sprang. It was quite a long time before I was willing to believe that it did not grow from the posterior wall of the vagina? My reason for this is because of the facet which I show you, which was proven to be the walls of the vagina, the mucous membrane going up the sides of the tumor, making it possible that it originated from the posterior wall of the vagina, or between the two layers, beneath the peritoneum and above the mucous surfaces, behind the uterus. The tumor may have first grown downwards pushing out the mucous membrane of the vaginal walls, then, owing to its increased size and the bony resistance of the pelvis extended upwards in the line of least resistance, pushing up Douglas pouch and making a hood for itself in this way. The lower part of the hood has been proven by microscopical examination to be the vaginal wall, but the upper part of the hood, which I also believe to be the vagina was not borne out by microscopical examination.

I also present for your examination several photographs of the tumor showing different views.

DR. W. O. ROBERTS: The specimen is exceedingly interesting.

I think it is similar to one I removed some time ago, Dr. Wathen being present at the operation. The tumor was fully as large as the one presented by Dr. Schachner and was found to spring from the posterior lip of the cervix. The uterus proper was not involved.

DR. W. C. DUGAN: I believe I am on record as stating that this tumor sprang from the posterior part of the cervix, in the space between the posterior wall of the vagina and Douglas pouch, in its growth separating the vaginal wall and lifting it upward. This is rather a common attachment for such tumors and most works on gynecological and abdominal surgery present cuts of just such tumors as Dr. Schachner has shown us. I will pass around the volume of Pozzi and here are two cuts, one showing the sub-peritoneal tumor like the one under consideration and the other the vaginal—the contrast is striking indeed.

DR. CARL WEIDNER: I simply wish to indorse what Drs. Schachner and Dugan have said. I do not think there can be any doubt about the tumor having arisen in the posterior part of the cervix. In its growth it pushed the vagina before it, and is to that extent an intra-vaginal tumor.

DR. W. H. WATHEN: The tumor evidently arose in the lower posterior part of the cervix. I have never seen, nor have I read the description of a fibroid tumor of this size arising in the vagina. Had it arisen within the vagina it would necessarily have grown downward; it would never have gone upward as this tumor did. It is a myomatous tumor arising in the lower posterior part of the cervix, lifting the peritoneum from the vagina and the rectum in Douglas pouch carrying it up in advance of the tumor. We find these retro-peritoneal and intraligamentous myomatous tumors quite frequently. I probably may have had a greater proportion than is customary, but I think at least one-fourth the cases I have seen have been of that character. Just what course they will take in separating the peritoneum, or in unfolding one or both broad ligaments, we can never say in advance. You will find in one case the broad ligaments are not unfolded. Where a tumor arises in the lower part of the cervix posteriorly it may lift the peritoneum and extend up under the sigmoid flexure of the colon, even unfolding the mesentery of the colon; the same tumor may extend up on the opposite side going under the cecum. So you may find the mesentery of the sigmoid flexure of the colon unfolded and the muscular tissue of the bowel lying directly on top of the tumor; or you may find the muscular tissue of the cecum occupying a similar position. I remember some years ago I operated on a case at St. Joseph's Infirmary,—Dr. Dugan assisted me,—where the mesentery of the colon was entirely unfolded. The same thing occurs in cystic tumors arising in the hilum of the ovary, involving the structures of the broad ligaments first, then taking up the peritoneum from Douglas pouch, or sometimes going in front, involving the broad ligament on one side, stripping the peritoneum from the anterior abdominal wall, so that you might make an incision and enucleate the tumor without having cut the peritoneum, or without entering the abdominal cavity. These tumors to me are very easily understood, and I think the more we study them the more easily we are able to understand them. Three or four years ago I began the earnest study of tumors of this class, including cystic growths, and have written several papers on the subject. I am sure Dr. Schachner will find that the specimen is simply a retro-peritoneal myomatous tumor. I recently operated upon a lady from New Albany, Ind., having a tumor similar to this, except that it was considerably larger. In that case the tumor had developed downward between the vagina and rectum until it came within one and a half inches of the orifice, the uterus being pushed up in front behind the pubes.

DR. AUGUST SCHACHNER: Dr. Wathen's remarks of to-night hardly agree with what he said at the previous meeting. I understand he agrees now that this tumor sprang from the posterior lip of the cervix, lifting the uterus upward, and also carrying with it a part of the vagina, and that it did not arise in the broad ligament.

